# Plasmid Replicons for the Production of Pharmaceutical-Grade pDNA, Proteins and Antigens by *Lactococcus lactis* Cell Factories

**DOI:** 10.3390/ijms22031379

**Published:** 2021-01-30

**Authors:** Sofia O.D. Duarte, Gabriel A. Monteiro

**Affiliations:** 1 iBB-Institute for Bioengineering and Biosciences, Instituto Superior Técnico, Universidade de Lisboa, Av. Rovisco Pais, 1049-001 Lisboa, Portugal; sofia.duarte@tecnico.ulisboa.pt; 2 Department of Bioengineering, Instituto Superior Técnico, Universidade de Lisboa, Av. Rovisco Pais, 1049-001 Lisboa, Portugal

**Keywords:** *Lactococcus lactis*, replicon, plasmid DNA, live mucosal vaccination

## Abstract

The *Lactococcus lactis* bacterium found in different natural environments is traditionally associated with the fermented food industry. But recently, its applications have been spreading to the pharmaceutical industry, which has exploited its probiotic characteristics and is moving towards its use as cell factories for the production of added-value recombinant proteins and plasmid DNA (pDNA) for DNA vaccination, as a safer and industrially profitable alternative to the traditional *Escherichia coli* host. Additionally, due to its food-grade and generally recognized safe status, there have been an increasing number of studies about its use in live mucosal vaccination. In this review, we critically systematize the plasmid replicons available for the production of pharmaceutical-grade pDNA and recombinant proteins by *L. lactis*. A plasmid vector is an easily customized component when the goal is to engineer bacteria in order to produce a heterologous compound in industrially significant amounts, as an alternative to genomic DNA modifications. The additional burden to the cell depends on plasmid copy number and on the expression level, targeting location and type of protein expressed. For live mucosal vaccination applications, besides the presence of the necessary regulatory sequences, it is imperative that cells produce the antigen of interest in sufficient yields. The cell wall anchored antigens had shown more promising results in live mucosal vaccination studies, when compared with intracellular or secreted antigens. On the other side, engineering *L. lactis* to express membrane proteins, especially if they have a eukaryotic background, increases the overall cellular burden. The different alternative replicons for live mucosal vaccination, using *L. lactis* as the DNA vaccine carrier or the antigen producer, are critically reviewed, as a starting platform to choose or engineer the best vector for each application.

## 1. Introduction

Lactic Acid Bacteria (LAB) constitute a heterogeneous group of Gram-positive bacteria that share the ability to produce lactic acid as a major product from the fermentation of sugars. They are non-spore forming, chemoorganotrophic bacteria and can occur as cocci or rods. LAB can be found in fermented foods and beverages, plants, sewage and also in the respiratory, genital and intestinal tract of humans and animals [1].

Phylogenetically, based on 16S and 23S sequence data, the Gram-positive bacteria can be divided in the *Clostridium* branch, with less than 50% of GC content, and in the Actinomycetes branch, with more than 50% GC content. The typical LAB belong to the low GC content branch and include *Carnobacterium*, *Lactobacillus*, *Lactococcus*, *Leuconostoc*, *Pediococcus* and *Streptococcus* genera [1].

Among *Lactococcus* genus, the non-pathogenic *L. lactis* is the best characterized LAB due to its wide applicability in industry (pharmaceutical, food, cosmetic and energy), mainly as a starter for cheese fermentation. It is also found on the surfaces of plants and in the gastrointestinal tract of animals, and is used as model for research in molecular biology, genetics, physiology and the metabolism of LAB [2]. Several *L. lactis* strains and some species of the *Lactobacillus* genus are used for biotechnology applications [3] due to their food-grade and Generally Recognized As Safe (GRAS) status. More recently, these LAB are used as producers and also as mucosal delivery vehicles of therapeutic molecules (plasmids or proteins), which contributed to raising the industrial relevance of these species [4].

*Escherichia coli* has been the major prokaryotic system used as a cell factory, due to its ability to produce very high levels of recombinant proteins and plasmids. Nevertheless, *E. coli* produces highly immunogenic lipopolysaccharides (LPS) [3,5], which emphasizes the importance and necessity of implementing *L. lactis* as a safer alternative to *E. coli*. At least one *E. coli* strain has a genetically modified LPS, which does not evoke an endotoxic response [6], but still lacks the food-grade and GRAS attributes. More specifically, *L. lactis* can be used as a cell factory for several different applications, such as the production of pharmaceutical- or food-grade proteins or metabolites with high yields for industrial application (e.g., lactic acid, ethanol, vitamins) [7], the production of pharmaceutical-grade plasmid DNA (pDNA) to use in DNA vaccination [8,9], or used as live vector for the delivery of DNA, proteins or metabolites to mucosal surfaces (i.e., live mucosal vaccination) [10].

The above referenced examples reinforce the potential of *L. lactis* to produce added-value compounds in an economically profitable manner, other than just lactic acid [11].

## 2. Plasmid Vectors Available for Plasmid DNA and Recombinant Protein Production by *L. lactis*

Many of the *L. lactis* applications as cell factory for high-yield production require its transformation with a plasmid harboring one gene (or more) of interest. The gene might encode a protein or an enzyme involved in the production of a metabolite (e.g., lactic acid, ethanol, vitamins). *L. lactis* can also be used for the production of pharmaceutical-grade pDNA, harboring a gene coding an antigen to be used in naked DNA vaccination. Additionally, *L. lactis* can be used in live mucosal vaccination, where bacterial cells are used as live vectors for the delivery of pDNA, proteins or metabolites to mucosal surfaces. The next sections will focus on the type of plasmid vectors used for each of these three different applications. Since the plasmid copy number (PCN) influences the pDNA yields and, consequently, the recombinant proteins yield, the origins of replication of *L. lactis* plasmids will be analyzed thoroughly.

### 2.1. Production of Pharmaceutical- and Food-Grade Proteins and Metabolites

The efficient protein secretion system of *L. lactis* confers to this species a major advantage as a protein production platform [7], unlike *E. coli* for which the most commonly used production strategies are intracellular (periplasm or cytoplasm), thus leading to expensive downstream purification processes. Additionally, unlike *E. coli*, *L. lactis* is devoid of the highly immunogenic LPS that may be co-purified with the proteins of interest and should be removed before administration to humans, making the manufacturing process more profitable and safe [12]. Concerning protein production, *L. lactis* is also an attractive alternative to the competitor Gram-positive bacterium *Bacillus subtilis*, the main drawback of which is the degradation of the secreted recombinant proteins by its complex extracellular proteolytic system [12]. *L. lactis* has only one major protein, Usp45, which is secreted into the medium, simplifying the downstream purification processes, and only has one housekeeping protease for secreted proteins, HtrA [12]a protease-free mutant [13]. Other Gram-positive bacteria, such as *Mycobacterium* or *Streptococcus*, have been studied as hosts for heterologous protein production [14], but the *L. lactis* food-grade and GRAS status turn them into a valuable alternative. Several heterologous proteins and metabolites have already been successfully produced in *L. lactis*, such as reporter proteins, bacterial, eukaryotic and viral antigens, interleukins, allergens, virulence factors, bacteriocins and enzymes with industrial relevance [7]. The main proteins and metabolites produced by *L. lactis* have already been extensively reviewed elsewhere [15]. Table 1 summarizes the main products made by *L. lactis* strains suitable for industrial production, together with the plasmids used to express the gene(s) or Open Reading Frame (ORF) of interest. The majority of the plasmids had a pSH71 replicon and further information about it will be provided latter.

### 2.2. DNA Vaccination 

The development of vaccines against several pathogens is one of the most important advances in medicine, as vaccines can eradicate diseases (e.g., smallpox) [32]. 

The progresses in the molecular biology field triggered the development of DNA or gene-based vaccines. This type of vaccine is able to trigger both cytotoxic (cellular) and antibody (humoral) immune responses against pathogens, without the safety issues of the live attenuated type [33]. DNA vaccines cause prolonged expression of the antigen, generating significant immunological memory, and for viral pathogens the antigens are expressed in the host on its natural form, which improves the immune response [10]. One of its main disadvantage is the low immunogenicity registered when used alone or without assisted delivery, and besides several clinical trials being approved, only DNA vaccines for the veterinary field were approved for commercialization [33]. Large doses of DNA vaccine (1–100 μg) are necessary for an effective induction of the host immune response [34]. Although there are potential risks associated with oncogene activation by DNA insertion into the host genome or associated with anti-DNA antibodies elicitation [35], several recent studies attribute to DNA vaccines a promising potential application in treating or preventing inherited and acquired diseases, such as cancer and viral infections [33].

DNA vaccines are composed of a pDNA vector that allows gene delivery into the nucleus of target cells, enabling the production of the desired protein (antigen) in situ. This has an enormous advantage when compared with the recombinant protein vaccination, where it is needed to deliver protein molecules, which are lost in huge amounts during trafficking to the target site. Several characteristics must be accomplished in order to consider DNA vectors appropriate for clinical application: (i) gene transcription in the cell must be ensured, (ii) gene degradation has to be avoided and (iii) safety must be guaranteed. From a commercial point of view, to be broadly applied, they should be inexpensive and easy to produce and purify, in high concentrations and quantity [36].

The vaccination efficiency can be improved by using chemical (e.g., micro and nanoparticles) or biological (viral or live bacteria) systems for the pDNA delivery [10,34]. The viral systems (retroviral, adenoviral and adeno-associated) are currently the most effective, in terms of DNA delivery to the nucleus and expression. However, this vector type raises several safety concerns regarding immunogenicity, toxicity, reversion to virulence, random integration into the genome and activation of oncogenes or deactivation of tumor-suppressing genes [10,37]. As a safer alternative, non-viral systems have been increasing their relevance, although they are still less efficient when compared with viral systems [10,36,38]. 

The use of bacteria as pDNA carriers brings some advantages, since bacteria are able to deliver the pDNA to the host cells, on one side, and help to improve the host immune response by its own immunogenic features [34]. Since pDNA-carrying bacteria are delivered through mucosas, is called live mucosal vaccination. This type of vaccines has the advantage of stimulating the mucosal immune system, besides the humoral and cellular components of the systemic immune system that other types of vaccines already stimulate (for a review, see [39]). LAB are ideal for this purpose, since they are non-invasive, GRAS and food-grade, being acceptable for administration through mucosal routes (e.g., oral, intranasal). Bacteria are able to protect the pDNA from degradation by the harsh environment inside the host mucosa [34].

The use of *L. lactis* as cells factories for naked pDNA production, as well as live carriers for mucosal vaccination, will be addressed in the following sections. 

#### 2.2.1. Naked DNA Vaccination and *L. lactis* as pDNA Producer

**Figure 1:**
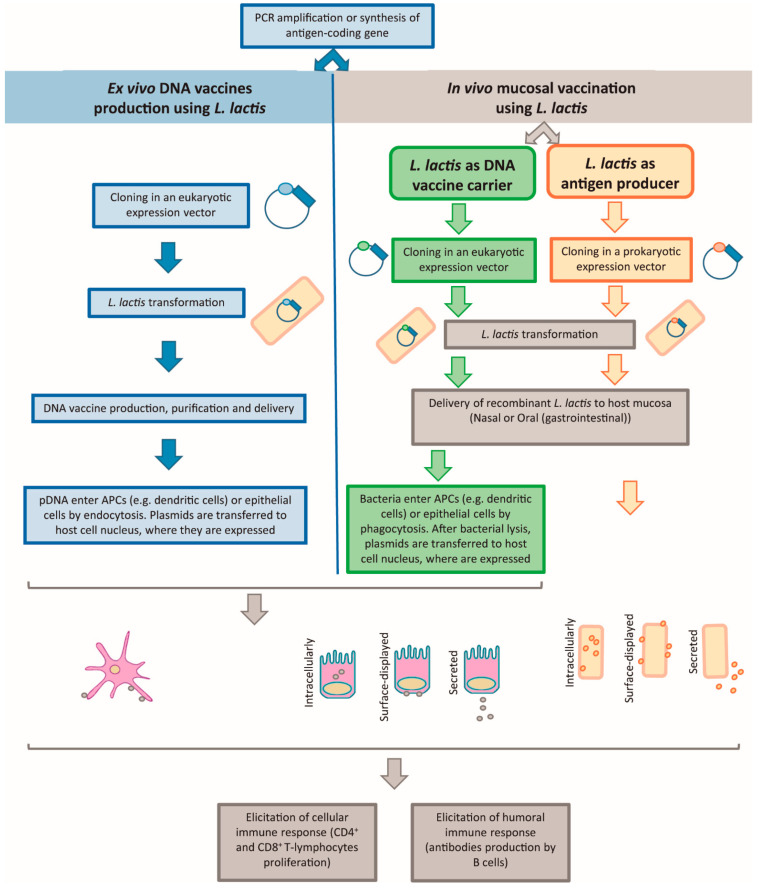
Comparison of the main steps of *Lactococcus lactis* being used as DNA vaccine producer or as delivery vector for mucosal vaccination either as a vaccine carrier or as an antigen producer [40,41,42,43,44].

Although the majority of studies with *L. lactis*-based DNA vaccination use *L. lactis* as the DNA vaccine carrier or as antigen delivery vector in live mucosal vaccination, there are several studies using engineered *L. lactis* as a cell factory to produce naked pDNA to be used as DNA vaccines (Figure 1). An interesting example of a DNA vaccine production study goes back to 2007, to a study where the authors constructed a plasmid expressing the HIV-1 gp120 protein. The intramuscular immunization of mice with pDNA produced by *L. lactis* developed gp120 antibody titers comparable to pDNA from *E. coli*, showing that *L. lactis* are a suitable host for DNA vaccines production, with the advantage of increased safety, when compared with LPS-producing *E. coli* [8,9].

**Figure 2:**
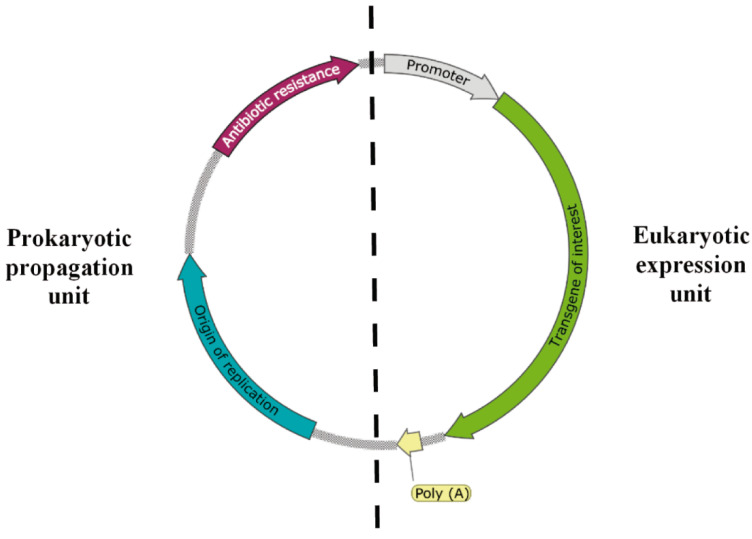
Essential elements of a plasmid DNA vector. The propagation unit should contain the antibiotic resistance gene and the bacterial origin of replication, for propagation and selection in bacteria. The expression unit should include the gene encoding the protein of interest, under the control of a ubiquitous (e.g., viral) promoter, and with transcription termination sequences (Poly A).

In order to use *L. lactis* as a cell factory to produce naked pDNA for DNA vaccination (Figure 1), cells are transformed with a plasmid vector containing both a prokaryotic replicative cassette and a eukaryotic expression cassette harboring the antigen gene of interest under the control of a eukaryotic promoter (Figure 2). Subsequently, *L. lactis* cells are grown at a large scale for pDNA production and purification. The purified DNA vaccine is then introduced into host eukaryotic cells (e.g., dendritic or epithelial cells) [41,42,45], by an appropriate delivery method (e.g., viral gene delivery systems, microinjection, gene gun, biojector, electroporation, ultrasound) [46]. After escaping the phagolysosome, the pDNA is transcribed, then the translated antigen can be presented (by using appropriate targeting signals) at the cytoplasm, at the cell surface or secreted. If the antigen is expressed at the cytoplasm of dendritic cells or another Antigen Presenting Cell (APC), the antigen is presented via MHCI and MHCII, being responsible for the elicitation of the cellular immune response (CD8^+^ and CD4^+^ T-lymphocytes proliferation). When epithelial cells or other non-APC cells are transfected, the antigen is processed and presented via MHCI. The secretion of the antigen to the host mucosa allows the protein to be bound to B-cell receptors, stimulating the humoral immune response, but can also be taken up by APCs, stimulating the cellular immune response, as explained above [41,42,45]. When the antigen is expressed at the cell surface, the antigen will mainly stimulate the humoral immune response.

#### 2.2.2. Live Mucosal Vaccination and *L. lactis* as DNA Vaccine Carrier or as Antigen Producer 

Live mucosal vaccination is a strategy where bacterial cells carrying antigen-coding pDNA are administered through host mucosal routes (Figure 1). *L. lactis* can be used just as a carrier, delivering the antigen-coding pDNA to the host mucosal cells where it will be expressed, or also as in situ antigen producer, delivering the antigen in the host mucosa and stimulating its immune system. The majority of the bacterial strains tested for mucosal vaccination were attenuated pathogens such as *Listeria monocytogenes*, *Salmonella typhi* and *Shigella flexneri*, due to their ability to naturally infect the mucosal surfaces. However, those species had a high risk associated with possible reversion to virulence, and therefore they are considered not entirely safe for use in humans, especially in children and immunosuppressed people. A harmless alternative was imperative and the research turned to LAB as safe mucosal DNA delivery vehicles [47]. More specifically, *L. lactis* had been extensively studied, due to its GRAS and non-pathogenic status, for antigen and cytokines production and delivery, as well as a vehicle for oral delivery of DNA vaccines [48]. important aspect is the fact these bacteria are able to survive through the gastrointestinal (GI) tract of humans for at least 2–3 days without evoking strong host immune responses [5,49], making them ideal to be used as live vectors for mucosal vaccination. 

##### *L. lactis* as a DNA Vaccine Carrier

Instead of vaccination with naked pDNA (Section 2.2.1.), which is prone to degradation at the harsh mucosal environment, the use of *L. lactis* to carry and protect pDNA is an alternative (Figure 1). Once at the host mucosa, the plasmids can be delivered to the eukaryotic host cells (dendritic cells or epithelial cells) after bacteria adhesion [50] followed by its internalization by phagocytosis [51,52,53,54]. The adhesion mechanism is partially characterized for *Lactobacillus* strains, being dependent on carbohydrate and protein factors on the bacterial cell surface [50]. More recent studies showed that the host intestinal cells have Toll-like and Nod-like receptors which recognize microbe-associated molecular patterns (MAMPs) present at the bacterial surface [34]. The internalization step by phagocytosis can occur both in phagocytes, such as dendritic cells, and also in epithelial cells, which could act as “non-professional” phagocytic cells [52]. The bacterial cell wall disruption with one of the several available chemical treatments increases significantly the success of the pDNA release inside eukaryotic cells, but it is not necessary to be an invasive strain, keeping intact its GRAS status [52]. These authors suggest different alternative mechanisms for the improved internalization of the cell wall weakened bacteria, related with a putative increase resistance of the intact bacteria to “non-professional” phagocytosis, the removal of inhibiting markers or with the exposure of phagocytosis-enhancing receptors recognizable by the epithelial cells.

Once inside the eukaryotic cell, the bacterial cells are lysed inside the phagolysosome, the pDNA containing a eukaryotic cassette is released and it enters the nucleus, where the gene of interest is transcribed by the host cells [53]. One example of a nuclear localization signal is the simian virus (SV)40 enhancer region. The transcription factors that bind this sequence in the cytoplasm have the ability of facilitating the pDNA active nuclear entry [41,55]. Then, the translated antigen can be presented (cytoplasm, surface-displayed or secreted) to the immune system (Figure 1, Section 2.2.1).

##### *L. lactis* as an Antigen Producer

Live mucosal vaccination using *L. lactis* to deliver the antigens requires a prokaryotic expression pDNA vector carrying the antigen gene being expressed in *L. lactis*, which is delivered to the host mucosa (e.g., nasal or oral/gastrointestinal). The antigen can be presented in one of three different ways: (i) cytoplasmic, which requires bacterial lysis for antigen release and delivery to the target cells, but has the advantage of protecting the antigen from degradation in the host mucosa; (ii) secretion to the host mucosa, where the antigen contacts directly with the mucosal epithelium and consequently the target cells; and (iii) cell surface expression, in which the antigen is anchored at the cell membrane, protecting it from proteolytic degradation [40,43,44]. Both cellular and humoral immune responses can be elicited in a similar manner as already explained before (Figure 1, Section 2.2.1). 

Advantageously, *L. lactis* is capable of expressing membrane-anchored heterologous proteins in much higher amounts than *E. coli* and *S. cerevisiae* (5–6% *versus* 1–1.5% and 0.5% respectively, of the total membrane proteins) [56]. The stability of both secreted and surface displayed proteins in *L. lactis* were compared in two recent studies. The plasmids used in these studies have pSH71-based replicons with the Usp45 signal peptide sequence for protein secretion fused with the gene of interest, and the addition of three LysM repeats in the surface-displayed version. The surface displayed proteins in *L. lactis* were considered more stable and with higher bioactivity than the secreted counterparts [57,58]. In a different study from Ma et al. [59], the authors administered to chicken live *L. lactis* expressing cytoplasmic, secreted or surface anchored *Eimeria tenella 3-1E* protein. Again, the surface anchored protein allowed the highest protection from *E. tenella* infection, since it elicited more effective immune responses. 

The yields of both cytoplasmic and secreted proteins were also compared in several different studies, with secretion allowing higher protein yields than cytoplasmic production [7]. Ribeiro et al. [60] used pWV01 and pAMβ1-based plasmids to express the *Brucella abortus* antigen L7/L12 in *L. lactis*. When the proteins where expressed at the cytoplasm, the maximum achieved yield was 0.5 mg/L, while the secreted counterpart achieved 3 mg/L. The fusion of the L7/L12 protein with the staphylococcal nuclease (Nuc) or with a synthetic propeptide (LEISSTCDA) increased the protein secretion yield to 8 mg/L. The proteins produced in the *L. lactis* cells cytoplasm are more susceptible to proteolysis from the Clp complex, while secretion seems to be an efficient way for proteins to escape proteolysis [7].

In sum, allowing the protein of interest to escape the cytoplasm, allows an increase in the protein production yield, while anchoring the protein at the cell surface increases its stability and immune response bioactivity. Several strategies to surface-display recombinant proteins in *L. lactis* were already reviewed elsewhere [61]. The most recent studies using *L. lactis* for mucosal vaccination (pDNA or antigen delivery systems) are summarized below in Table 2, Table 3 and Table 4. The plasmids used to express the intended antigen in this species are also presented and can be grouped in three main replicons that will be described in further detail: pWV01- and pSH71-based replicons with a rolling-circle (RC) replication mechanism and the pAMβ1-based replicon, which has a theta-type mode of replication. Each replicon has a different PCN in *L. lactis* cells, which influences the plasmid and recombinant proteins yields. These considerations are of utmost importance when the final goal is to use these recombinant strains to perform live mucosal vaccination in humans or in animals, where it is crucial to guarantee that the highest amount of antigen could be achieved. 

##### Plasmids with a pWV01-Based Origin of Replication

**Figure 3:**
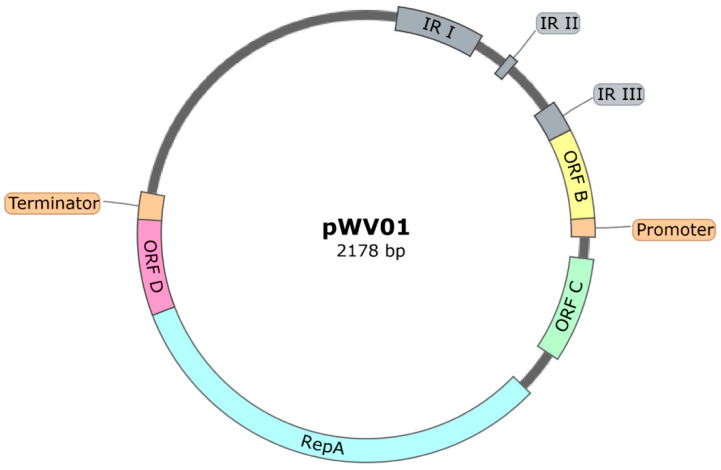
Main features of the plasmid pWV01 replicon. The *repA* and *orfC* genes code for the replication initiation protein and for a protein involved in negative copy number control, respectively. The Open Reading Frame ORFD is putatively involved in copy number regulation, but the ORFB function is still not clear. The double-strand origin (DSO) necessary for the rolling-circle (RC) replication process is located in the inverted repeat (IR) III, while the single-strand origin (SSO) is located in the IR I and IR II. Compiled based on data from [62].

The pWV01-based origin of replication originally derives from the 2.3 kb pWV01 cryptic plasmid isolated from *L. lactis* subsp. *cremoris* Wg2. It has a RC mode of replication and the replication initiator protein is the 27 kDa RepA. The replication of the RC plasmids proceeds via single-stranded intermediates and its conversion to the double-stranded form initiates at the single-strand origin (SSO), by a RNA polymerase-independent process. There are four groups of RC plasmids, distinguished based on similarities in the leading strand replication region. The pWV01 plasmid belongs to the pE194/pLS1 group, which has a double-strand origin (DSO) in the inverted repeat (IR) III, an SSO in the IR I and IR II, a *repA* gene that codes for the replication protein that introduces a single-strand nick in the plus origin, a *repC* (*orfC*) gene coding for a 6 kDa protein involved in negative copy number control and a *repC/repA* terminator (Figure 3) [62]. There is another ORF, called ORFD, putatively involved in copy number regulation [63]. Due to the presence of several copy number control mechanisms, the pWV01-based vectors show a low PCN (3-10 plasmids per cell, determined by scintillation) [64,65,66]. 

The pWV01 origin of replication has been used to create more than 20 different cloning vectors. pGK12 is one of the first pWV01-based plasmids, replicating in a wide range of hosts, including *B. subtilis*, *E. coli*, *L. lactis* and several *Lactobacillus* species, but it is unstable and has a low copy number in these species (3, 5 and 60 copies per *L. lactis*, *B. subtilis* and *E. coli* cell, respectively) [63,65]. A high copy number pWV01-derivative (pBAV1K-T5) was engineered by deleting repeats IV to VI and the ORFD, leading to 68 and 251-357 copies per cell in *B. subtilis* and *E. coli*, respectively [63]. Nevertheless, pWV01 is widely used as a cloning vector [66,67] and there are several recent examples of pWV01-based plasmids being used for DNA and mucosal vaccination (Table 2). 

The main pWV01-based plasmids used in the most recent works for live mucosal vaccination were pValac, pPERDBY, pSEC (able to secret the coding antigen proteins), pCYT (express antigen proteins that remain at the cytoplasm) and pMG36e. 

**Figure 4:**
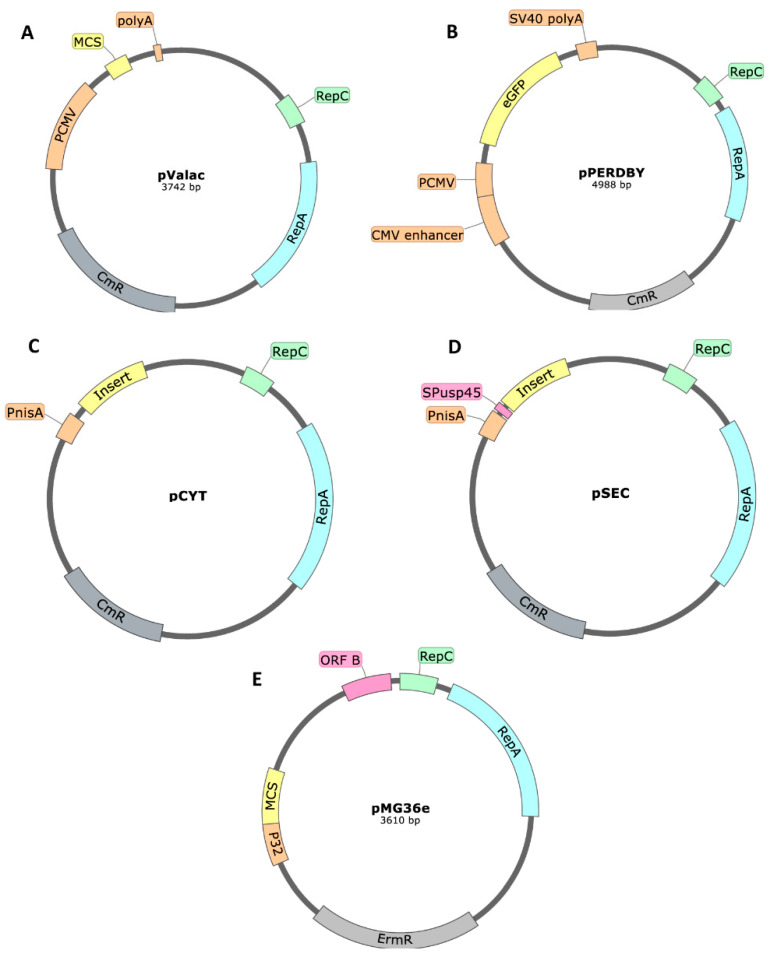
Structure of the pWV01-based plasmids (**A**) pValac [51], (**B**) pPERDBY [54], (**C**) pCYT [94], (**D**) pSEC [94] and (**E**) pMG36e [90]. The plasmids generally contain the replication origin genes (*repC* and *repA*), the antibiotic resistance gene (chloramphenicol, *cm^R^*, or erythromycin, *erm^R^*) and the gene of interest under the control of a eukaryotic (the cytomegalovirus promoter, P_CMV_) or prokaryotic promoter (inducible, such as P_nisA_ or constitutive, such as P_32_). The eukaryotic expression plasmids also have a polyadenylation region (polyA).

Both pValac (Figure 4A) and pPERDBY (Figure 4B) plasmids were designed with the goal of using non-invasive *L. lactis* as a DNA vaccine carrier. The pPERDBY is similar to pValac with the additional cloning of the enhanced Green Fluorescence Protein (eGFP), under the control of a CMV promoter and with a SV40 early mRNA polyadenylation signal, as well as immunostimulatory CpG motifs as an adjuvant. Similarly to pValac, pPERDBY is able to replicate both in *E. coli* and LAB, being one of the smallest reporter plasmids developed for DNA vaccination, with less than 5 kb [54]. CHO-K1 and Caco-2 cells were efficiently transfected with pPERDBY after delivery by co-cultured recombinant non-invasive *L. lactis* cells [54]. Later, in 2017, the Internalin A (InlA) of *Listeria monocytogenes* gene was added to the construction and the study showed that the invasive strain resulted in a three-fold increase in the number of Caco-2 cells expressing the eGFP. The InlA invasion protein binds to the E-cadherin receptor expressed on human epithelial cells, allowing a more rapid and efficient entry by endocytosis of the *L. lactis* bacteria and consequently an enhanced DNA delivery to the nucleus [54]. The same has already been accomplished with pValac [51]. In 2019, pPERDBY evolved to the vaccine platform LacVax^®^ and the authors showed that *L. lactis* transformed with LacVax^®^ expressing the Outer Membrane Protein A (OmpA) of *Shigella dysenteriae* type-1 is able to induce a systemic and mucosal immune responses in a murine model for shigellosis, after oral immunization [84]. Another strategy to turn *L. lactis* into an invasive strain is to modify it in order to express the fibronectin-binding protein A (FnbpA) of *Staphylococcus aureus*. The FnbpA mediates the invasivity of *L. lactis* in nonphagocytic host cells, improving its ability to deliver DNA. Zurita-Turk et al. [72] successfully used this invasive strain to deliver interleukin-10 (IL-10) to IL-10 deficient mice, using pValac as the gene-carrying plasmid.

When the goal is to use recombinant *L. lactis* in live mucosal vaccination, pCYT (Figure 4C), pSEC (Figure 4D) or pMG36e (Figure 4E) plasmids could be a suitable choice. The pCYT plasmid is an integrative plasmid for cytosol expression, while pSEC is an integrative plasmid for secretion expression [94]. For the antigen to be expressed at the cell surface, instead of being secreted, an alternative is to clone the antigen coding gene (ORF) in a pSEC plasmid as a fusion protein together with LcsB that has the ability to anchor the antigen at the cell surface [75]. Both pCYT and pSEC have the P_nisA_ prokaryotic inducible promoter regulating the gene of interest. The origin of replication and antibiotic resistance gene are the same as in pValac [94]. The additional feature present in pSEC plasmid is a signal peptide of *usp45* (*SPusp45*) from *L. lactis*. The pMG36e plasmid has two major differences from the remaining prokaryotic expression vectors: the antibiotic resistance gene confers resistance to erythromycin, instead of chloramphenicol, and the gene of interest is under the control of the constitutive promoter P32, instead of an inducible one.

A study from Carmen et al. [73] compared the efficacy of using *L. lactis* as a DNA vaccine carrier or as an antigen producer, in colorectal cancer prevention. The antigen (IL-10) was delivered both as cDNA or protein produced by the genetically modified LAB and the results showed that the cDNA delivery was less effective in reducing cancer incidence in a colorectal cancer mouse model. The plasmid used for the IL-10 cDNA expression was pValac, while pGroESL (pSEC plasmid-derived) was used for the IL-10 protein production by an expression system inducible by stress (SICE) [73].

A recent study used recombinant *L. lactis* harboring a pSEC plasmid containing the *Mycobacterium leprae* heat shock protein 65 (Hsp65) gene under the control of a xylose-inducible promoter. The continuous production of Hsp65 in the gut by the recombinant strain, after oral treatment, prevented induced arthritis in mice [82].

A study used two different plasmids to harbor the necessary antigens: the original pCYT with the inducible promoter P_nisA_, and the pCYT-based pHJ plasmid with the constitutive promoter P32. The recombinant *L. lactis* strains were able to successfully deliver the antigens (heat shock protein 65 and tandemly repeated IA2P2) to the intestinal mucosa, while protecting the proteins from degradation inside non-obese diabetic (NOD) mice. The antigens could be detected in the duodenal and ileum mucosa as long as five days after recombinant *L. lactis* administration and efficiently prevented type 1 diabetes mellitus in NOD mice [86]. This study showed that *L. lactis* is able to survive in the host mucosa for an extended time, while continuously producing the antigen of interest, independently of the type of promoter used.

Zhou et al. [91] used the pMG36e plasmid both for intracellular or extracellular *Taenia solium* TSOL18 protein production. For protein secretion, the gene was cloned downstream of a signal peptide SP_Usp45_ or in fusion with propeptide LEISSTCDA. The secretory recombinant strains showed protein expression both in the extracellular supernatant and in the intracellular precipitation, while in its cytosolic counterpart it was only detected intracellularly. The antibody levels, intestinal mucosa-specific sIgA levels, spleen lymphocyte proliferation levels and cytokines levels were higher in mice immunized with the secretory recombinant *L. lactis*, being more efficient in vaccination against cysticercosis than the cytoplasmic version of the plasmid.

##### Plasmids with a pSH71-Based Origin of Replication

The pSH71 replicon from the 2.1 kb *L. lactis* subsp. *lactis* 712 plasmid is closely related to the pWV01 replicon, also having the *repA* and *repC* genes and replicating by a RC mode. It is also able to replicate both in Gram-positive and Gram-negative bacteria. Several cloning plasmids were engineered from the pSH71 replicon, such as the high copy number pCK1, the high copy number pNZ12 and the low copy number pNZ121 [95]. The PCN for the parental pSH71 was estimated as 200 copies per LAB cell [96].

The pSH71 is very similar to pWV01, differing only in a few nucleotides and in the absence of a direct repeat. Like pWV01, the plasmid pSH71 has a high segregational stability in *L. lactis* and other Gram-positive hosts, with a plasmid loss being less than 10^−5^ per generation, but a low stability in *E. coli* (loss of more than 10^−2^ plasmids per generation) [64].

**Figure 5:**
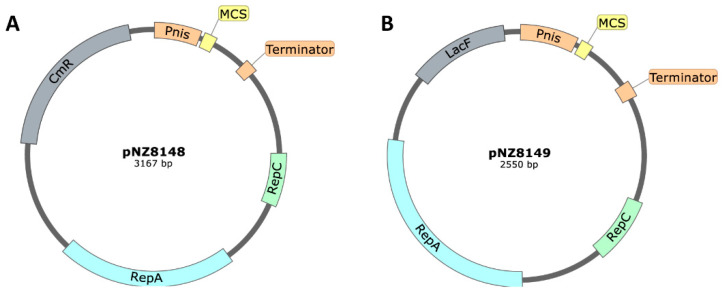
Map of (**A**) pNZ8148 [98] and (**B**) pNZ8149 [99]. The plasmids generally contain the replication origin genes (*repC* and *repA*), a selection marker (chloramphenicol resistance gene, *cm^R^*, or a food-grade marker, *lacF*) and the gene of interest under the control of an inducible prokaryotic promoter (P_nisA_).

The main pSH71-derived backbones plasmids used in live mucosal vaccination studies with *L. lactis* were pNZ8148, pNZ8149 (Figure 5), pNZ8150, pNZ8048 and pLZ12km2. The pNZ8148 (Figure 5A) plasmid contains the nisin A promoter (P_nisA_) followed by a Multiple Cloning Site (MCS) and downstream of the MCS it contains a terminator. This broad-host-range vector has resistance to chloramphenicol and a replication origin with the replication genes A and C (*repA* and *repC*). The only difference in pNZ8149 (Figure 5B) is that it has the *L. lactis lacF* gene as food-grade selection marker, instead of the antibiotic resistance gene. For transformants selection, this vector needs a host strain with the lactose operon without the *lacF* gene, such as *L. lactis* NZ3900. The pNZ8150 is very similar to pNZ8148, with the difference that the translation fusions are performed at the ScaI site, downstream of the ATG. pNZ8048 is identical to pNZ8148, except in the presence of additional 60 bp in pNZ8048 that corresponds to residual *B. subtilis* DNA that were deleted during pNZ8148 construction [97]. pNZ8110, pNZ8124 and PNZYR are very similar to pNZ8148, with the addition of signal sequence of the major secreted protein Usp45 of *L. lactis* upstream of the MCS, for protein secretion. pNZ8121 is also a vector optimized for protein secretion, but with the signal sequence of PrtP instead of Usp45. The pLZ12km2 plasmid is an *E.coli*/streptococcus shuttle vector containing a constitutive P23 lactococcal promoter and a kanamycin resistance gene. There are several examples of live mucosal vaccination studies using *L. lactis* carrying pSH71-derived plasmids (Table 3).

The development of a vaccine against bird flu is a good example of expressing a haemagglutinin antigen intracellularly, from a pSH71-based plasmid in *L. lactis*, instead of being secreted, to protect the antigen from the passage through the stomach. The *H5* gene was cloned under the control of the nisin-controlled gene expression system in the pNZ8150 plasmid and the preliminary results showed that the oral delivery of live *L. lactis* cells producing H5 protein was able to elicit an immune response in chicken and mice [118]. A more recent example of a recombinant antigen being expressed in a soluble form in *L. lactis* cytoplasm was accomplished by Wang et al. [105]. The authors engineered *L. lactis* with a pNZ8149 plasmid for nisin-induced expression of the fusion protein containing the RCK (Resistance to complement killing, responsible for bacteria internalization) protein of *Salmonella enterica* and VP2 (major antigen) of infectious bursal disease virus (IBDV). After oral or injected administration of the inactivated recombinant *L. lactis* to chickens, a specific neutralizing-antibody-mediated immune response was observed against an IBDV challenge. Song et al. [117] also used a similar strategy to co-express intracellularly in *L. lactis* the fusion antigen STa-LTB-STb-F5 of enterotoxigenic *E. coli* (ETEC) and the outer membrane protein (Omp) H of the M cell-targeting ligand of *Yersinia enterocolitica*. Oral immunized mice showed a complete protection after an ETEC challenge, due to an increase in the production of CD4^+^ and CD8^+^ T-cells, lymphocyte proliferation and secretion of cytokines. These studies showed that intracellularly expressed antigens are able to induce mucosal, humoral and cell-mediated immunity.

Secretion of antigens can be achieved by cloning the signal sequence of *usp45* gene upstream of the antigen coding gene (ORF) in the pNZ8149 plasmid. Recently, Sun et al. [102] used this approach with *L. lactis* for producing heat-labile enterotoxin B subunit as adjuvant in oral vaccines formulation. When co-administered to mice with a *Helicobacter pylori* vaccine candidate expressing Lpp20 antigen, it significantly enhanced the mucosal antibody responses against *H. pylori* [102]. A different study used a recombinant *L. lactis* with a pNZ8048 vector expressing exendin-4, a receptor agonist that is a therapeutic peptide drug for type 2 diabetes. The Usp45 signal peptide and LEISSTCDA propeptide were concatenated with the exendin-4 sequence to guarantee an efficient secretion by *L. lactis*. INS-1 cells treated in vitro with recombinant exendin-4 secreted by *L. lactis* significantly enhanced insulin secretion and showed enhanced proliferation and inhibited apoptosis [120]. A more recent example of a secreted antigen come from Namai et al. [149]. The authors used the plasmid pNZ8148#2:SEC (pNZ8148 plasmid with a Usp45 sequence) to clone the IL-1 receptor antagonist (IL-1Ra) gene and express it in *L. lactis*. The recombinant bacterial strain was orally administered to mice and significant levels of IL-1Ra were detected in the mice colon, where it inhibited the IL-1 signaling and alleviated the acute colitis symptoms. These results proves that recombinant *L. lactis* are able to reach the colon alive and secrete IL-1Ra in situ. The authors also showed that IL-1Ra translocated to the blood after being secreted into the colon mucosa. In this study, IL-1Ra was secreted in a much higher amount (2 mg/L) when compared with other cytokines in previous studies. The same plasmid was used in a pioneer study [148] that showed that recombinant *L. lactis* can be administered nasally in order to deliver therapeutical molecules to the lungs, and further into the systemic circulation.

To enable antigen anchoring to cell surface, the antigen coding gene (ORF) could be fused to the cell wall anchoring motif *lysM* and to the secretion signal of the lactococcal Usp45 protein [100]. With this system, mucosal (oral) vaccination with *L. lactis* expressing *H. pylori* adhesin A (HpaA) using pNZ8110 evoked an immune response against *H. pylori* [100]. A similar strategy was used by others using pSH71-based vectors (pMEC237) for recombinant *L. lactis* live mucosal vaccination [167]. A different strategy was adopted by Joan et al. [119], consisting in the pandemic H1N1 2009 haemagglutinin 1 antigen fused to the nisP anchor protein being expressed in *L. lactis* using pNZ8048 plasmid and given to mice as an oral vaccine. The authors have shown that this vaccine was able to elicit the humoral immune response of BALB/c mice; even the HA1 epitope was not detected at the bacterial cell surface [119]. A more recent study using surface displayed antigens was developed by Lahiri et al. [121], where pNZ8048 was used to display the ectodomain of influenza matrix protein 2 and neuraminidase in the *L. lactis* surface. The specific strategy to achieve the membrane anchoring of the antigens was to clone the genes between an upstream signal peptide of *L. lactis* protease Usp45 and a downstream cell wall anchoring motif from *Streptococcus pyogenes* (CWAM6). This recombinant strain have a great efficiency in inducing mucosal and systemic immune responses in chicken, against pathogenic avian influenza virus infection. Recently, alternative strategies to display the antigens at the *L. lactis* surface were developed, such as using a combination of Usp45 and the cA (C terminus of the peptidoglycan-biding domain) domain of the N-Acetyl-muraminidase [145], the Spax anchoring domain (Lei et al., 2020) or the PrtP signal peptide [161].

Two main studies tried to compare the efficacy of cytoplasmic, secreted and surface displayed antigens in developing strong immune responses against different diseases. Li et al. [125] used pTX8048 (a pNZ8048 derived plasmid) to express cytoplasmic, secreted (encoding signal peptide of secretion protein Usp45) and cell-wall anchored (encoding Usp45 and cell-wall anchor region with LPXTG-type anchoring motif) EtAMA1 (Apical 16 membrane antigen 1 of *E. tenella*) in *L. lactis*, as a solution for avian coccidiosis. After the recombinant strain being given orally to chickens, all the immunized animals showed immune protective effects, but especially the chickens that were given the cell-wall anchored bacteria. The authors suggested that proteins expressed in the cell wall might be more resistant to degradation when compared with the secreted and cytoplasmic ones. The cell-wall proteins will hence interact in a more efficient manner with the intestinal M cells or dendritic cells (antigen presenting cells), being presented by histocompatibility complex class I molecules, inducing the activation of CD4^+^ T helper cells. Similar results were obtained by Wang et al. [126] using the same plasmid vector. The authors showed that cell-wall anchored antigens evoked stronger immune responses when compared with cytoplasmic or secreted ones. Škrlec et al. [134] compared the production yield of secreted and surface displayed antigens. Although the previous studies showed improved results for the surface displayed antigens, Škrlec et al. [134] showed that the secretion strategy led to higher yields than the cell-wall anchored strategy (117 vs. 30 ng/mL).

###### Plasmids with a pAMβ1-Based Origin of Replication

The pAMβ1 plasmid was isolated from *Enterococcus faecalis* and replicates by a unidirectional theta mechanism. This replicon has a high structural stability that allow cloning large fragments and has a wide host range within Gram-positive bacteria [169]. The highest number of copies reported for the pAMβ1 replicon was around 100 copies per *B. subtilis* cell, after inactivation of the transcriptional repressor *copF* that represses the plasmid-encoded replication initiation protein RepE [169]. Both *repD* (unknown function) and *repE* genes are under the control of the P_DE_ promoter and they have their transcription, and ultimately the PCN, tightly regulated by two different systems: the transcriptional repressor protein CopF and an antisense RNA-mediated transcription attenuation system [170].

**Figure 6:**
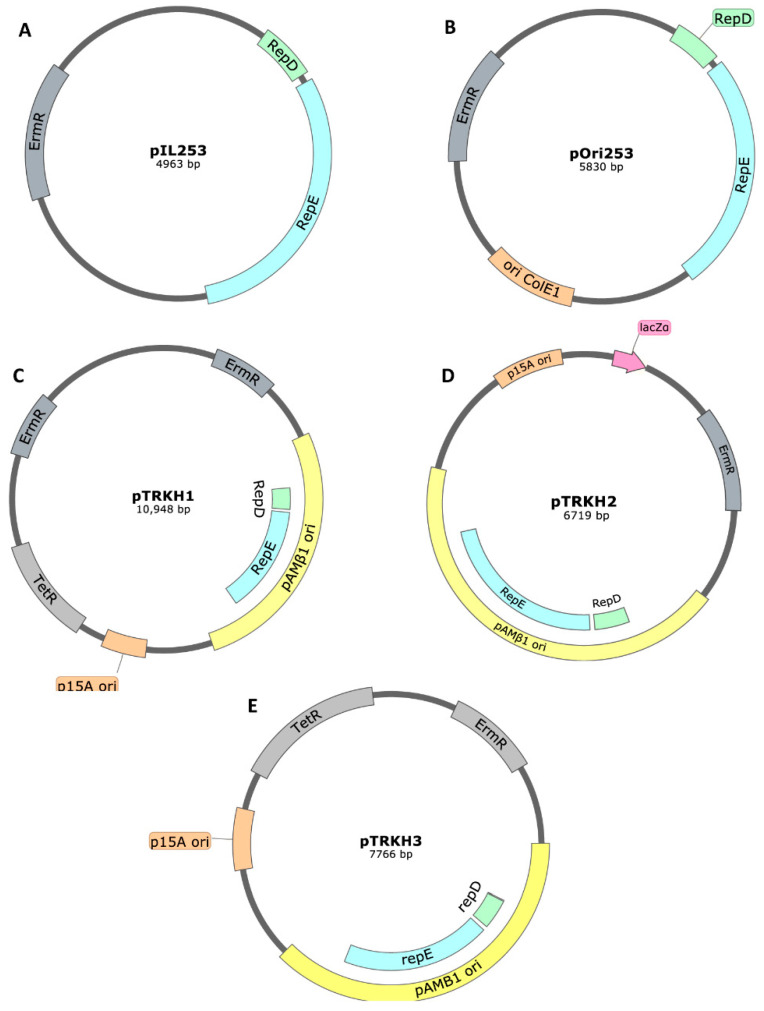
Map of (**A**) pIL253 [19], (**B**) pOri253 [19], (**C**) pTRKH1 [17,18], (**D**) pTRKH2 [17,18] and (**E**) pTRKH3 [17,18] plasmids. The plasmids generally contain the pAMβ1 replication origin genes (*repD* and *repE*) and an antibiotic resistance gene (erythromycin, *erm^R^*, and in some plasmids tetracycline, *tet*^R^). The most recent plasmids also harbor a Gram-negative origin of replication (ColE1 or p15A).

The main pAMβ1-based plasmids used in mucosal vaccination using *L. lactis* as a host were derived from pIL253 (Figure 6A). This vector has an erythromycin resistance gene and the pAMβ1 origin of replication, with *repD* and *repE* genes that replicates in Gram-positive hosts. It is able to replicate at high copy number (45–85 copies per cell, determined by densitometry) [171] and is able to stably maintain large DNA inserts [19]. Several *L. lactis* mucosal vaccination studies were performed used pAMβ1-derived backbone plasmids with promising results (Table 4) [172,173,174].

However, since pAMβ1-derived plasmids are unable to replicate in *E. coli*, the research has turned to the creation of pAMβ1-based shuttle vectors, by cloning other origins of replication in the same plasmid. From the pIL253 backbone (Figure 6A), three main shuttle vectors, able to replicate in Gram-positive and Gram-negative hosts, were engineered. The addition of the Gram-negative replicon is of utmost importance, since it allows the molecular cloning techniques to be performed in a simpler and faster way using the well-studied model organism *E. coli.* The Gram-positive bacteria can then be transformed with the final plasmid, avoiding more time- and resource-consuming techniques that are necessary for applying molecular biology techniques with Gram-positive hosts. The pOri253 plasmid (Figure 6B) was constructed by insertion of the *ColE1* origin of replication from Gram-negative bacteria in the pIL253 MCS [186]. After the insertion of the *P23* lactococcal promoter, the new expression vector was called pOri23 [19].

The pTRK family of shuttle vectors was also derived from pIL253 after insertion of a Gram-negative p15A origin of replication, resulting in the pTRKH1 vector (11 kb) (Figure 6C), which also harbored the tetracycline resistance gene for selection in Gram-negative hosts (erythromycin resistance works in both Gram-negative and Gram-positive hosts). The pTRKH1 PCN in Gram-positive bacteria was similar to the parental plasmid and in *E. coli* it had a medium copy number (30–40 plasmids per cell). pTRKH2 plasmid (6.9 kb) resulted from the improvement of the pTRKH1 vector, after incorporation of a *lacZ* cassette and removal of non-essential Gram-negatives sequences (Figure 6D). The pTRKH2 plasmid allowed a blue/white screening in *lacZα*-complementing *E. coli* strains, turning the cloning process less time consuming. Its reduced size should also contribute to a more efficient transformation of the *L. lactis* strains. Also from pTRKH1, the more recent pTRKH3 (7.8 kb) (Figure 6E) was constructed after removal of non-essential sequences, remaining with erythromycin and tetracycline resistance genes and both origins of replication (pAMβ1 for Gram-positive and p15A for Gram-negative hosts) [17,18]. It has around 30–40 copies per *E. coli* cell and a high copy number (45–85) in streptococcal and lactococcal hosts [187]. The pAMJ328 plasmid is quite similar to pTRKH2, with the addition of the P170 promoter, which is pH-inducible and growth phase-dependent [188]. pAMJ2008 is similar to pAMJ328, but with the additional secretion signal SP310mut2* that is able to generate the native N-terminus on the secreted recombinant protein. pAMJ399 has an identical secretion signal, but that leaves an extension of four amino acids AERS on the secreted recombinant protein.

There are several recent examples of pAMβ1-based plasmids used to transform *L. lactis* with the goal of being DNA vaccine carriers or live vectors for mucosal vaccination. An example of the first application can be found in a recent study using *L. lactis* for pDNA delivery to eukaryotic cells using pOri253 as backbone, where the results showed that CHO cells were able to express the GFP reporter protein as early as 12 h after bacterial inoculation [176]. The authors developed a new plasmid pExu, derived from pOri253, containing a theta-type origin of replication and an expression cassette containing the *eGFP* gene under the control of a CMV viral promoter. *L. lactis* transformed with the former construction were administered by gavage to Balb/C mice and the mice enterocytes showed eGFP protein expression. Additionally, an in vitro experiment showed that 15.8% of CHO cells were able to express the protein after transfection, meaning that *L. lactis* harboring the pExu plasmid is an excellent candidate for gene delivery to eukaryotic cells, to be used as a live vehicle for DNA vaccines delivery [176].

As happened with the aforementioned replicons, the majority of the studies with pAMβ1-based vectors address the use of *L. lactis* for live mucosal vaccination, with antigens being expressed intracellularly, anchored at the cell surface or secreted to the host mucosa. Aliramaei et al. [179] cloned the *H. pylori cagL* gene, which expresses a highly conserved protein located at the tip of a pili, into the pAMJ2008 vector. Although pAMJ2008 is a secretory vector, the expression of the protein was only detected intracellularly. Even with this setback, the recombinant strain was orally administered to mice, being able to stimulate CagL specific antibodies. In the same year, Wang et al. [181] used the similar secretory vector pMJ399 to harbor the porcine circovirus type 2 capsid protein and showed that *L. lactis* were able to efficiently secrete it. The recombinant strain was able to survive the gastrointestinal tract of mice during at least 11 days, after oral administration, while inducing mucosal, cellular and humoral immune responses against porcine circovirus type 2 infection. A recent example of a surface displayed antigen expressed by a pAMβ1 vector is the study from Derakhshandeh et al. [183]. The authors used the pT1NX vector containing the lactococcal Usp45 secretion signal sequence, the sequence encoding the cell wall anchor of *S. aureus* protein A (*spaX*) and the antigen of interest (FimH) under the control of the constitutive P1 promoter. The recombinant *L. lactis* were administered to mice bladders where it efficiently protected against urinary tract infections caused by uropathogenic *E. coli*.

## 3. Conclusions: Choosing or Developing/Constructing the Best Vector for Each Application

The data analyzed in the present review give an important insight about the more appropriate *L. lactis* vectors to choose or how to engineer them when the goal is to produce high amounts of pDNA and/or recombinant protein, with high quality standards for pharmaceutical or industrial applications.

One of the most important characteristics to consider when choosing a vector is its type of replication origin, since it will influence the PCN, which in turn affects the *L. lactis* pDNA and recombinant protein production.

There are three main replicons available for *L. lactis*: pWV01, pSH71 and pAMβ1. Although the pWV01-type vectors have a low PCN (<15 copies), they have a broad host range, being able to replicate both in Gram-positive and Gram-negative bacteria. There are several vectors available with a pWV01 origin of replication, such as pValac (PCN = 15) ([189], determined by real-time quantitative PCR), pMG36e (PCN = 5) [190], pPERDBY, pCYT and pSEC.

The pSH71 replicon is quite similar to pWV01, only differing in a few nucleotides, but has a much higher PCN, around 200 copies per cell [96]. It is also able to replicate both in Gram-positive and Gram-negative bacteria, having a high segregational stability in Gram-positive hosts, but a low stability in *E. coli* [64]. There are a huge variety of pSH71-based vectors, with components necessary for cytoplasmic, secretory or cell-wall anchored antigen expression.

The pAMβ1 plasmids have a high structural stability that allows cloning large fragments and has a wide host range within Gram-positive bacteria. The highest number of copies calculated for the pAMβ1 replicon was around 100 copies per *B. subtilis* cell [169]. pIL252-derived vectors have a low PCN of 6–9 copies per chromosome, while pIL253 derivatives (pOri253 and pTRK family vectors) have 45–80 copies per chromosome [187,191].

Historically, the production of pharmaceutical- or food-grade proteins and metabolites was accomplished using pSH71-based vectors, due to its higher PCN and consequently its high protein yield. However, a good example of DNA vaccine production in *L. lactis* used a pAMβ1 replicon, which had a lower PCN value [8]. All live mucosal vaccination strategies depend on antigen production, which could be targeted to the cytoplasm, expressed at the cell surface or secreted to the extracellular medium [44]. There are examples with plasmids harboring each of the three types of replicons (pWV01, pSH71 and pAMβ1) in all types of live mucosal vaccination studies, with a higher incidence with the pSH71 replicons (for the complete reference list, see Table 2, Table 3 and Table 4).

Overall, for pDNA production by *L. lactis* to be used as DNA vaccines it should be desirable to use a vector that allows reaching the highest PCN possible, such as a pSH71-based vector. For bulk protein production, the rationale is the same, since a high PCN linearly correlates with a high yield of protein production.

For live mucosal vaccination applications, one should account for the impact in the cell growth and metabolism, since it is necessary that cells stay alive for a longer period of time, while producing the antigen of interest. The cell wall anchored antigens had shown more promising results in live mucosal vaccination studies, when compared with intracellular or secreted antigens. However, engineering *L. lactis* to express membrane proteins, especially if they have a eukaryotic background, increases the overall cellular burden. More specifically, the overproduction of membrane proteins leads to a cell envelope stress response, corresponding to an up-regulation of several chaperones and proteases to deal with the accumulation of misfolded proteins [192]. As a consequence, the expression of recombinant membrane proteins has an impact in several different processes, such as transcription, translation, targeting, membrane insertion and folding [192].

One could hypothesize that a medium PCN vector, such as the pIL253 derived vectors (pAMβ1 replicon), could be a logical choice to have acceptable pDNA and protein amounts, and at the same time preserve the metabolism and integrity of the *L. lactis* cells.

Choosing the best vector for a specific application could be to engineer a pre-existing vector in order to become suitable for each purpose, using appropriate synthetic biology tools. It is possible to increase the vector PCN by using different strategies. The simpler strategy is to simply change the entire origin of replication to a pSH71-based origin. The PCN of vectors harboring different types of origin of replication could be increased by deletion of auxiliary factors such as *rep* genes transcriptional repressors [169,193]. Alterations in the RBS sequence upstream the *rep* genes, in order to increase RBS strength or the probability of *rep* genes expression, could be an alternative solution, which has already been applied to a pAMβ1-based vector [191]. Finally, the entire promoter controlling the expression of the replication genes could be changed to a stronger one.

When the goal is to use a vector in live mucosal vaccination studies, it will be desirable to express the antigen at the cell surface, to increase its efficacy, in terms of antigen stability and immune response bioactivity. There are several strategies available to accomplish that goal, such as cloning the Usp45 signal peptide sequence for protein secretion fused with the gene of interest, together with the addition of three LysM repeats for surface-display [57,58]. The Usp45 signal peptide can be concatenated with the LEISSTCDA propeptide to increase the secretion efficiency by *L. lactis* [120]. Further alternatives to anchor the antigen to the cell wall were already reviewed elsewhere [194]. In live mucosal vaccination studies, it could also be important to have the gene coding for the antigen of interest under the control of an inducible promoter, instead of a constitutive one, for in situ controlled expression.

The modification in the plasmid vector could only increase its efficacy to a certain extent, it being important also to focus on strain modification, in order to improve its suitability for each application. More studies are needed to investigate ways to engineer the *L. lactis* genome, in order to overcome the hurdles found in the recombinant cell wall anchored proteins and to increase its efficiency and profitability in live mucosal vaccination applications.

## Figures and Tables

**Table 1:** Main proteins and metabolites suitable for industrial production by *Lactococcus lactis*.

Product	Plasmid	Replicon	Reference
L-alanine	pNZ2650 (pNZ8020 derived)	pSH71	[16]
Linalool	pNZ7640 (pNZ8150 derived)	pSH71	[17]
Germacrene D	pNZ8048	pSH71	[18]
β-Sesquiphellandrene	pNZ8048	pSH71	[19]
Hyaluronic acid	pSJR6 (pNZ8048 derived)	pSH71	[20]
Folate (vitamin B11)	pNZ7011 (pNZ8048 derived)	pSH71	[21,22]
Mabinlin II	pNZ8149	pSH71	[23]
β-Cyclodextrin glucanotransferase	pNZ8048	pSH71	[24]
Coumarate CoA ligase (4CL)	pFI2413	pSH71	[25]
Alcohol acyltransferase (SAAT)	pNZ7630 (pNZ8150 derived)	pSH71	[17]
Sesquiterpene synthase	pNZ8048	pSH71	[18]
3-Hydroxy-3-methylglutaryl CoA reductase (HMGR)	pNZ8048	pSH71	[19]
Bile salt hydrolase (BSH)	pNZ8149	pSH71	[26]
Acid urease	pNZ8148/pNZ8149	pSH71	[27]
Acetoin/diacetyl	pCIT264	Rep264	[28]
Lactic acid	-	-	[29]
Riboflavin (vitamin B2)	-	-	[30]
Ethanol	-	-	[31]

**Table 2:** Plasmids with a pWV01 origin of replication used as antigen-expressing vectors in *L. lactis* host, for mucosal vaccination purposes against several diseases.

Disease	Antigen/Cytokine	pWV01 Based-Plasmid	Reference
Tuberculosis	*Mycobacterium tuberculosis* ESAT-6	pValac	[10,68]
Tuberculosis	*Mycobacterium tuberculosis* Ag85A	pValac	[69]
Inflammatory bowel disease	IL-4	pValac	[70]
Inflammatory bowel disease	Anti-Tumor Necrosis Factor-alpha	pValac	[71]
Inflammatory bowel disease	IL-10 of *Mus musculus*	pValac	[72]
Colorectal cancer	Catalase, superoxide dismutase or IL-10	pValac/ pGroESL (pSEC derived)	[73]
*Shigella dysenteriae* diarrhea	Outer membrane protein A of *S. dysenteriae*	pSEC	[74]
Oral vaccines against cancer or infectious diseases	Carcinoembryonic antigen	pSEC	[75]
Inflammatory bowel disease	*Mycobacterium* heat shock protein 65 (Hsp65)	pSEC	[76]
Bovine tuberculosis	*Mycobacterium leprae* Hsp65	pSEC	[77]
Hemorrhagic shock	Heme oxygenase-1	pSEC	[78]
Intestinal mucositis	Human pancreatitis-associated protein I	pSEC	[79,80]
Oral vaccines against infectious diseases	*Staphylococcus aureus* nuclease fused with OmpH β1α1 domain of *Yersinia* *enterocolitica*	pSEC	[81]
Arthritis	*Mycobacterium leprae* Hsp65	pXylT:SEC (pSEC derived)	[82]
Allergic asthma	*Mycobacterium* Hsp65	pXylT:SEC (pSEC derived)	[83]
Shigellosis	Outer membrane protein A	pPERDBY (LacVax^®^, pSEC derived)	[84]
DNA vaccination	Enhanced Green Fluorescence Protein (EGFP)	pPERDBY (pSEC derived)	[53,54]
*Streptococcus iniae* infection	*S. iniae* M-like protein antigen	pCYT	[85]
Type 1 diabetes mellitus	Hsp65 and tandemly repeated IA2P2	pCYT	[86]
Type 1 diabetes mellitus	Staphylococcal nuclease (SNase)	pCYT	[87]
Fish edwardsiellosis	OmpA and flagellar hook protein D antigens from *Edwardsiella tarda*	pCYT	[88]
Inflammatory bowel diseases	Pancreatitis-associated protein	pSEC and pCYT	[89]
Gastrointestinal lead poisoning	Human metallothionein-I fusion protein	pMG36e	[90]
Cysticercosis	*Taenia solium* TSOL18 antigen	pMG36e	[91]
Hand, foot, and mouth disease	Viral protein 1 of enterovirus 71	pMG36e	[92]
Alzheimer’s and Parkinson’s diseases	Glucagon-like peptide-1	pMG36e	[93]

**Table 3:** Plasmids with a pSH71 origin of replication used as antigen-expressing vectors in *L. lactis* host, for mucosal vaccination purposes against several diseases.

Disease	Antigen/Cytokine	pSH71 Based-Plasmid	Reference
*Helicobacter pylory*	*H. pylori* adhesin A (HpaA)	pNZ8110	[100]
*H. pylory*, allergic diseases and carcinomas	*H. pylori* neutrophil-activating protein A subunit	pNZ8110	[101]
*H. pylory* vaccine adjuvant	Heat-labile enterotoxin B subunit	pNZ8149	[102]
*H. pylori* infection	*H. pylori* Lpp20 antigen	pNZ8149	[103]
Infectious bursal disease virus (chickens)	OmpH of an M cell-targeting ligand and IBDV-VP2 protein	pNZ8149	[104]
Infectious bursal disease virus (IBDV) (chickens)	Antigen VP2 of IBDV and RCK protein of *Salmonella enterica*	pNZ8149	[105]
*Proteus mirabilis*	OmpA of *P. mirabilis*	pNZ8149	[106]
Brucellosis	*Brucella melitensis* bp26 gene	pNZ8149	[107]
*Streptococcus pyogenes* infections	M-protein antigens derived from Group A *Streptococcus*	pNZ8149	[108]
*Bordetella pertussis* infection	F1S1 fusion protein (N-terminal region of S1 subunit from pertussis toxin and filamentous hemagglutinin type 1 immunodominant domain)	pNZ8149	[109,110]
Porcine epidemic diarrhea	Porcine Epidemic Diarrhea Virus S1Gene	pNZ8149	[111]
Duck hepatitis A virus (DHAV)	VP1 protein of DHAV type 3	pNZ8149	[112]
Cancer	Azurin	pNZ8149	[113]
*Campylobacter jejuni* infection	*C. jejuni cjaA* gene	pNZ8149	[114]
Obesity and diabetes	Fibroblast growth factor 21	pNZ8149	[115]
Atherosclerosis and nonalcoholic fatty liver disease	Ling Zhi 8 protein	pNZ8149	[116]
Enterotoxigenic *Escherichia coli*	Trivalent enterotoxin protein STa-LTB-STb and the F5 fimbrial antigen with OmpH of *Yersinia enterocolitica*	pNZ8149	[117]
Bird flu H5N1	Hemagglutinin from an Avian H5N1 isolate	pNZ8150	[118]
H1N1	H1N1 2009 haemagglutinin1 and nisP anchor fusion protein	pNZ8048	[119]
Type 2 diabetes	Exendin-4 (Exd4)	pNZ8048	[120]
Pathogenic avian influenzavirus infection	Ectodomain of influenza matrix protein 2 and neuraminidase	pNZ8048	[121]
Bovine tuberculosis	*Mycobacterium bovis* antigens MPB70 and MPB83	pNZ8048	[122]
*C. jejuni* infection	*C. jejuni* surface lipoprotein A	pNZ8048	[123]
Avian coccidiosis (*Eimeria tenella* infection)	*E. tenella* 3-1E protein	pTX8048 (pNZ8048 derived)	[59,124]
Avian coccidiosis (*E. tenella* infection)	Apical membrane antigen 1 of *E. tenella*	pTX8048 (pNZ8048 derived)	[125]
Hepatitis-splenomegaly syndrome in chickens	Avian hepatitis E virus truncated ORF2 protein	pTX8048 (pNZ8048 derived)	[126]
Thrombosis	Subtilisin QK-2	pRF01 (pNZ8149 derived) and pRF03 (pNZ8048 derived)	[57]
Diabetes	Single-chain insulin (SCI-59) analog	pMRF5018 (pNZ8149 derived) and pMRF5019 (pNZ8048 derived)	[58]
Human colon carcinoma	Bioactive kisspeptin (KiSS1)	pNZ401 (pNZ8048 derived)	[127]
Shiga Toxin 1 B subunit, from Shiga toxin-producing bacteria, like enterohemorrhagic *E. coli* and *Shigella dysenteriae*	Albumin-binding domain variants	pNZ8148	[128]
Inflammatory diseases, autoimmune diseases and cancer	Single-chain variable fragment antibody against mouse interleukin 6	pNZ8148	[129]
Inflammatory bowel diseases	Porcine insulin-like growth factor I	pNZ8148	[130]
House dust mite allergy	House dust mite allergen	pNZ8148	[131]
Liver detoxification	CYP3A4 and two isoforms of MGST1	pNZ8148	[56]
Japanese cedar pollinosis	Gene encoding fused T cell epitopes from the Cry j 1 and Cry j 2 antigens	pNZ8148	[132]
*Enteropathogenic E. coli* K.1.1 and human cervical carcinoma (HeLa) cells	Signal peptide gene (*plnA*) and bacteriocin encoding gene, plantaricin E (*plnE*)	pNZ8148	[133]
Gastrointestinal inflammation	Pentadecapeptide BPC-157	pNZ8148	[134]
Ulcerative colitis	Interleukin (IL)-35	pNZ8148	[135]
Inflammatory bowel disease	IL-23 Receptor-Targeted REX Protein Blockers	pNZ8148	[136]
Shiga toxins of *E. coli* O157:H7 and *S. dysenteriae*	Shiga toxin Stx2a1	pNZ8148	[137]
*E. coli* O157 and *Shigella flexneri*	Intimin and IpaB	pNZ8148	[138]
Cancer	Anti‑human cytotoxic T lymphocyte-associated antigen 4 Single‑Chain Fragment Variable	pNZ8148	[139]
H9N2 AvianInfluenza Virus (chickens)	M1 and HA2 proteins derived from an antigenically conserved endemic H9N2 virus strain	pNZ8148	[140]
Ulcerative colitis	Human defensin-5	pNZ8148	[141]
Foot-and-mouth disease	Foot-and-mouth disease virus VP1 gene	pNZ8148	[142]
Viral haemorrhagic septicaemia (fish)	G gene of viral hemorrhagic septicaemia virus	pNZ8148	[143]
H5N1	H5N1 infuenza virus haemagglutinin	pNZ8148	[144]
Spring viremia of carp virus (fish)	Spring viremia of carp virus glycoprotein	pNZ8148	[145]
Allergies	Bioactive anti‑interleukin‑4 single‑chain fragment variable	pNZ8148#2	[146]
Sepsis	Recombinant bovine lactoferrin	pNZ8148:CYT	[147]
Emphysema	Heme Oxygenase-1	pNZ8148#2:SEC	[148]
Inflammatory bowel disease	Interleukin 1 receptor antagonist	pNZ8148#2:SEC	[149]
Hirame novirhabdovirus	Hirame novirhabdovirus (HIRRV) G protein	pSLC (pNZ8148-derived)	[150]
Group A Streptococcus (GAS)	Fibronectin-collagen-T-antigen (FCT)-FCT-3 and FCT-4	pLZ12km2	[151]
Peptide vaccine	GAS serotype M1 pilus (PilM1) encoded in the FCT-2 region and Ova324–339	pilVax (pLZ12km2 derived)	[152]
*Staphylococcus aureus* infection	Linear B-cell epitope, D3(22–33), from the fibronectin-binding protein A of *Staphylococcus aureus*	pilVax (pLZ12km2 derived)	[153]
Human papillomavirus type 16-induced tumors	E6 oncogene	pNZ8123	[154,155]
HPV-16	Codon optimized E7 oncogenes isolated from 30 Iranian HPV-16	pNZ8148/pNZ8123	[156,157,158,159,160]
Cutaneous leishmaniasisis	PpSP15-EGFP	pNZ8121	[161]
Non-bacterial acute gastroenteritis	VP1 gene of a Human Norovirus GII.4	pNZ8150	[162]
Group B *Streptococcus* infections	Surface immune protein from Group B *Streptococcus*	pNZ8124	[163]
Colorectal cancer	Tumoricidal human tumor necrosis factor-related apoptosis-inducing ligand	pNZ8124	[164]
Brucellosis	Omp31 Antigen of *Brucella melitensis*	pNZ7021	[165]
Allergy to *Amaranthus retroflexus* pollens	Ama r 2 gene	pNZ7025	[166]
*Yersinia pseudotuberculosis* infection	LcrV from *Y. pseudotuberculosis*	pMEC237 (pNZYR derived)	[167,168]

**Table 4:** Plasmids with a pAMβ1 origin of replication used as antigen-expressing vectors in *L. lactis* host, for mucosal vaccination purposes against several diseases.

Disease	Antigen/Cytokine	pAMβ1 Based-Plasmid	Reference
*Campylobacter jejuni* infection	*C. jejuni* antigens	pIL253	[174]
Multiple sclerosis	Myelin peptides	pIL253	[173]
Colitis	cDNA of Microbial Anti-inflammatory Molecule (MAM) from *Faecalibacterium prausnitzii*	pILMAM (pIL253 derived)	[172]
HPV-16-induced tumors	HPV-16-E7 antigen	pOri23 (pIL253 derived)	[175]
DNA vaccine	eGFP	pExu (pOri253 derived)	[176]
Malaria	*Pfs*230 and *Pfs*48/45	pLEA2 (pAMJ328 derivative)	[177]
Malaria	Epitopes of key malaria parasite antigens: glutamate-rich protein (GLURP), merozoite surface protein 3 (MSP3), and the highly disulphide bonded Pfs48/45 (10C)	pSS3 (pAMJ328 derivative)	[178]
*H. pylory* infection	*H. pylori cagL* gene	pAMJ2008	[179]
Brucellosis	Omp16-IL2 fusion protein antigen	pAMJ2008	[180]
Porcine circovirus type 2 (PCV2)	Cap protein of PCV2	pAMJ399	[181]
Diabetes	Human proinsulin and IL-10	pT1NX (pTREX derivative)	[182]
Urinary tract infections	Uropathogenic *E. coli* FimH	pT1NX (pTREX derivative)	[183]
Parasitic worms	*Bacillus thuringiensis* crystal protein Cry5B	pTRK593 (pTRKH2 derived)	[184]
Rheumatoid arthritis	Murine IL-35	pMSP3535H3	[185]

## Data Availability

No new data were created or analyzed in this study.
